# *Leptospermum petersonii* as a Potential Natural Food Preservative

**DOI:** 10.3390/molecules25235487

**Published:** 2020-11-24

**Authors:** Wasiu Olalekan Afolabi, Ahmed Hussein, Francis Oluwole Shode, Marilize Le Roes-Hill, Fanie Rautenbach

**Affiliations:** 1 Department of Chemistry, Cape Peninsula University of Technology, Bellville 7535, South Africa; mohammedam@cput.ac.za; 2 Department of Biotechnology and Food Technology, Durban University of Technology, Durban 4000, South Africa; francisshode@gmail.com; 3 Applied Microbial and Health Biotechnology Institute, Cape Peninsula University of Technology, Bellville 7535, South Africa; leroesm@cput.ac.za (M.L.R.-H.); rautenbachf@cput.ac.za (F.R.)

**Keywords:** *Leptospermum petersonii*, 6-methyltectochrysin, antioxidant, antimicrobial, natural food preservatives

## Abstract

*Leptospermum petersonii* (family *Myrtaceae*) is often cultivated for ornamental purposes but also serves as a rich source of bioactive essential oils. While several studies focused on the activities of the essential oils, this study analysed the potential of spent *L. petersonii* leaves as a natural food preservative. Method: We investigated the in vitro antioxidant and antimicrobial activities of crude *L. petersonii* extracts against activities of the purified isolated flavonoid, 6-methyltectochrysin, which was characterized using spectroscopic methods. The antioxidant assays followed ORAC, FRAP and TEAC tests. The antimicrobial activities of the extract and purified flavonoid were analysed against six multi-drug resistant microbial strains in broth dilution assays. Result: The results revealed that both the crude extracts and isolated 6-methyltectochrysin exhibited positive radical ion scavenging antioxidant potential, however the crude extract was about 6-fold more potent antioxidant than the purified 6-methyltectochrysin. The crude extract also showed strong antimicrobial activities against *Bacillus cereus*, and even more potent antimicrobial agent than the reference ampicillin antibiotic against *Klebsiella pneumoniae* subsp. *pneumoniae.* A higher resistance was observed for the tested Gram-negative strains than for the Gram-positive ones. 6-methyltectochrysin was generally inactive in the antimicrobial assays. Conclusion: The crude methanolic extract showed significant bioactivity which validates the medicinal relevance of the plant. The observed biological activities, especially against a notorious strain of *B. cereus,* suggest that *L. petersonii* could be a promising natural source of food preservatives.

## 1. Introduction

Natural products have been used in folk medicine since ancient times. These naturally occurring compounds are often considered priori harmless [1] and may be sourced from plants or microbes. *Leptospermum* plant species have reportedly provided significant benefits in folk medicine. Ethnobotanical survey revealed that *Leptospermum flavescens* is used in Malaysian folk medicine to improve appetite, treat stomach disorders and relieve menstrual pain [2], while the essential oils from *L. scoparium* are widely used in New Zealand as an antibacterial agent [2,3]. The use of *Leptospermum* in the treatment of wounds/skin diseases was highlighted by Robson and Cooper [4].

Although *L. petersonii* is widely cultivated for its ornamental purposes, the essential oils extracted from the plant are potent biological agents [5], e.g., exhibiting remarkable antifungal activities, for instance, the effectiveness in the treatment of fungal infections in the mammalian lungs was reported by Hood et al. [6]. The essential oils from *L. petersonii* also exhibited bioactivities both in the in vitro and in vivo studies against *Aspergillosis* spp. and *Candida albicans* [7]. According to the authors, the neral and geranial constituents of the essential oils could be linked to the fumigant antifungal properties of the oils.

The increase in the incidences of antibiotic resistance remain a global persistent threat to human health [8]. Among the known multidrug-resistant pathogens is *Bacillus cereus*, an aerobic rod-shaped Gram-positive bacterium that leads to fried rice disease syndrome in Africa and elsewhere [9]. Fried rice syndrome caused by bacterial infestation with *B. cereus* has been implicated in food-borne diarrhea and may lead to severe soft tissue infection in other instances [9]. This is a phenomenal problem in regions where rice is a stable food, for example, in most underdeveloped countries. The poor food preservation and hygiene habits at food preparations spaces, and especially at ready-to-eat food vending spots create thriving hotspots for pathogens including *B. cereus* [10], which may lead to food poisoning. Natural food preservatives may thus proffer manageable solutions in such regions.

Antioxidants are biologically useful compounds that may help reduce the progression, or avoid oxidation of substrates. These compounds can inhibit or eliminate oxidative stress and damages to cells or other molecules within the body of living hosts. The neuroprotective properties of natural antioxidants are reported elsewhere [11]. Living organisms rely on antioxidant defense mechanisms provided by natural compounds, which are either produced within the body, for instance, in the synthesis of superoxide dismutase, or obtainable externally from nutritive diets. External (exogeneous) source of antioxidants (vitamins A, C and E, carotenes, lycopene, etc.) are mainly from plants. Antioxidants are believed to serve as protection against tissue damage in plants, but in humans, dietary intake of antioxidants has several benefits, including a decline in the proliferation of cardiovascular diseases, cancer, and aging [12].

Flavonoids or flavonoid-containing products are reported to possess good antioxidant activities [13,14]. Flavonoids are secondary metabolites widely distributed in the leaves, barks, seeds, bark and flower of several plants and may be found in human diet such as fruits, vegetables, wines, teas and cocoa [15]. The increasing interest in studying flavonoids lies in their antioxidant and chelating abilities which serve as protection against various diseases, including cardiovascular and carcinogenic diseases. The free radical suppressing activities are attributed to their chemical structures [16] that include one hydroxyl group, double bonds and carbonyl functionality.

The food industry often utilizes food-grade antioxidants for two main purposes: (i) prevent reduction in quality of food products and (ii) to preserve the nutritional composition and value of food products [17]. There is an increasing global focus on natural functional or dietary foods from plant sources. Phytomedicinal compositions are well suited for these needs. Polyphenolic compounds are finding useful applications as food preservatives [18] due to the global appeal for the substitution of synthetic preservatives in food products. In this study, we report preliminary data for the potential exploitation of spent *L. petersonii* leaves as an agri-food waste product which may serve as a phytomedicinal source that could aid human wellness and find applications as a natural food preservative.

## 2. Results and Discussion

### 2.1. Extraction Yields

From the sequential extraction scheme illustrated and discussed in the methodology section, the product yields are as follows: essential oil (12 mL); oily/gelatinous material (18 g); yellowish solid (2.4 g); recrystallized flavonoid (0.64 g); marc 2 residue (12 g). The recovered yield of the isolated flavonoid was about 0.24% of the raw leaves.

### 2.2. Characterization of Isolated 6-methyltectochrysin

A portion of the yellowish powdery material obtained (0.6 g) was subjected to silica gel column chromatography to obtain pure yellowish crystals (235 mg, one spot on TLC) (suspected flavonoid) while other fractions obtained were (suspected) mixtures of flavonoid and triterpenes. The purified compound, yellowish needle-shaped crystals had R_f_ 0.72 (hexane: ethyl acetate 8:2) and a melting point of 214 °C. The mass spectral analysis (ESI-MS [M−H]-) for the compound was *m/z* 281.098 (calc. 282.29), corresponding to the molecular formula C_17_H_14_O_4_.

FT-IR (*v*_max_ cm^−1^): 3065 (-OH), 3004 (allylic C=CH), 2922 (CH), 2852 (CH), 1660 (carbonyl C=O), 1611, 1590 (Ar-), 1493, 1450, 1381, 1333, 1133.

**Figure 1:**
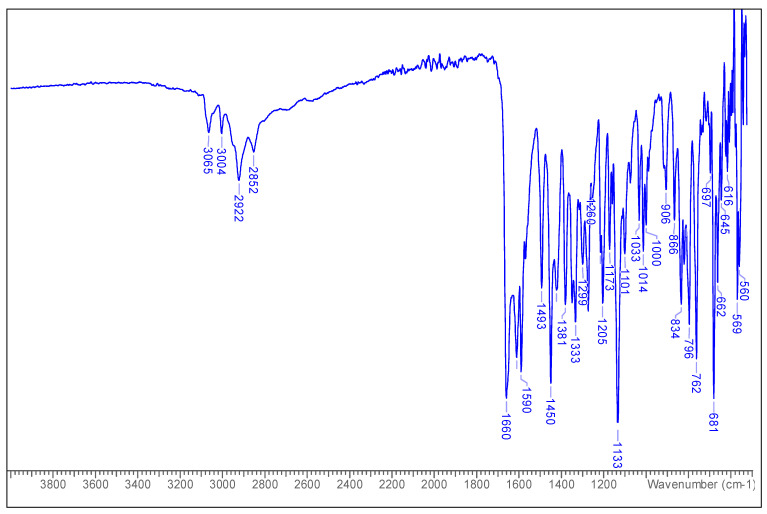
FT-IR spectrum of 6-methyltectochyrin.

The IR data of the compound (Figure 1) showed strong prominent carbonyl (C=O) absorption at 1660. The absorption bands at 1590 depict aromatic rings, while 3004 represent allylic C=CH stretch. The absorption peaks at 2922 and 2852 indicated the presence of CH groups.

^1^H-NMR (600 MHz, CDCl_3_) δ ppm: 1.23 (1H, s); 2.27 (3H, s); 3.88 (3H, s); 6.38 (1H, s); 6.64 (1H, s); 7.51 (3H, m); 7.88 (2H, m).

**Figure 2:**
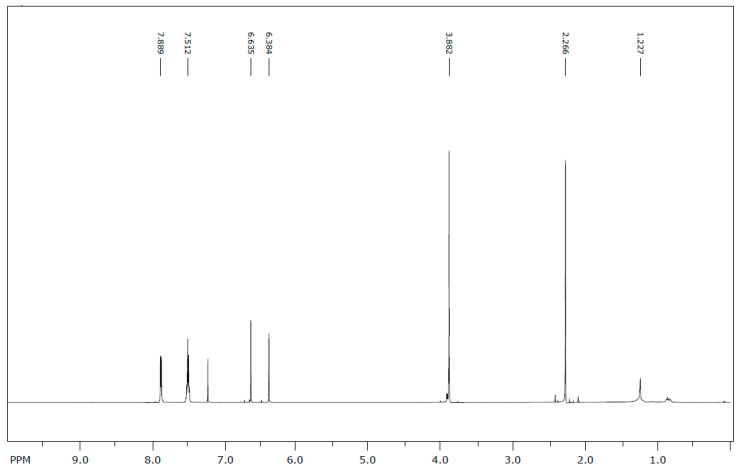
^1^H-NMR spectrum of 6-methyltectochrysin.

The ^1^H NMR spectrum (Figure 2) revealed aromatic protons at 7.88 (2H) and 7.51 (3H), and other protons (ring B) at 6.64 (H–3) and at 6.38 (H–8). The 7–OMe and 6–Me protons were assigned at 3.88 and 2.27, respectively [lit. 3.96 (7–OMe), 2.13 (6–Me)] [19].

**Figure 3:**
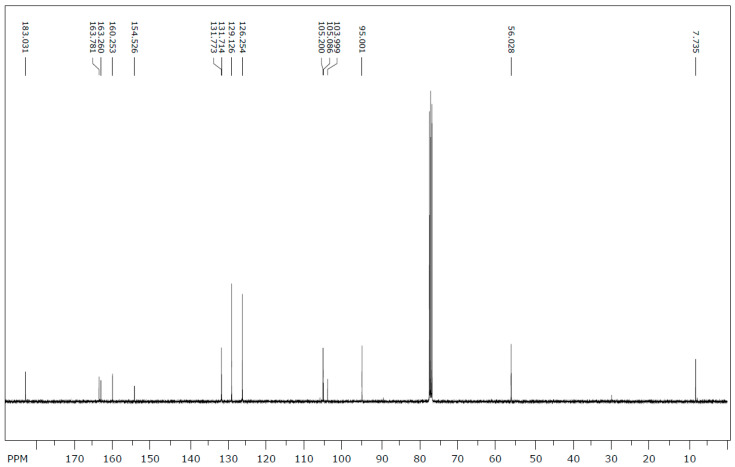
^13^C-NMR spectrum of 6-methyltectochrysin.

The ^13^C NMR data were extrapolated from the NMR spectrum (Figure 3). The results and carbon assignment are presented in Table 1. The results were analysed for the presence and the relative positions of specific functional groups, e.g., the hydroxyl, the methyl and the methoxy functionalities to generate the final structural. From the NMR and mass spectroscopic analyses (as well as data from literature), the isolated flavonoid was identified as 5-hydroxy-7-methoxy-6-methylflavone or 6-methyltectochrysin.

### 2.3. Antioxidant Activities

The ORAC assay examines the peroxyl radical absorbing capacity of the flavonoid-containing extract and the purified flavonoid while the TEAC assay informs the potential of antioxidants to scavenge ABTS radical cation. The FRAP assay assesses the ability of antioxidant compounds to reduce Fe^3+^ to Fe^2+^ by donating electrons. These assays have been used to determine the antioxidant capacity of the compounds of interest and the capacities are presented in Table 2.

Both the crude extract and the flavonoid compound (6-methyltectochrysin) isolated from *L. petersonii* showed positive antioxidant activity. The crude extract of *L. petersonii* was found to exhibit better antioxidant activity than the purified isolated flavonoid. The oxygen radical absorption of the crude extract was found to be 2-fold greater than 6-methyltectochrysin, and about 8-fold higher in ferric-reducing potential relative to the isolated flavonoid. The ABTS radical scavenging assay showed a similar trend, with the methanolic crude exhibiting almost 3-fold higher antioxidant capacity than the isolated flavonoid. The purified flavonoid and the naturally occurring antioxidant samples had lower antioxidant capacities against the ascorbic acid control.

The differences in the antioxidant capacities of the extract relative to the purified flavonoid may suggest the influence of other components present in the crude extracts towards a higher antioxidant activity. For instance, *Leptospermum* species are known to contain a mixture of terpenes such as 1, 8-cineole and terpinen-4-ol [20]. Other studies supported the observation that some crude extracts often possess higher antioxidant activities than their purified isolates [21] and that the combined effect of compounds in crude extracts may help to improve the overall antioxidant activities [22].

The Oxygen Radical Absorbance Capacity (ORAC) assay combines both the time and levels of inhibition in a phase result calculated by the area under the curve generated in the time vs. degree of inhibition plot [23]. This study employed the use of AAPH as the oxidant source which produces radical ions required to mimic generation of oxidative radicals in vivo. The ORAC peroxyl radical results of the antioxidant capacities of the crude extract and purified flavonoid exhibited some degree of antioxidant capacity, though the activity was improved for the crude extract. Previous structure-activity-guided studies have indicated that the phenolic –OH functionalities of flavonoids influence the radical scavenging properties of flavonoids [24]. The hydroxyl and methoxy groups and their relative positions in the flavonoid structure also influence the iron radical antioxidant activities and the TEAC (ABTS+) radical scavenging potential.

It was initially suggested that the flavonoid constituent of *Leptospermum* species might be responsible for its medicinal properties given its wide application in folk medicines as a sedative, and in the treatment of respiratory infections and diarrhea [25], however, this study has shown that, although the flavonoid exhibited levels of antioxidant activities, there may be other constituents of the crude extract which may influence the overall biological activities. It is, however, noted that the total phenol content (TPC) and total flavonoid content (TFC) should be determined and correlated with the results of the antioxidant assays to corroborate this hypothesis in further studies.

### 2.4. Antimicrobial Assays

In vitro broth dilution assays were carried out against six species of multi-drug resistant pathogenic bacteria strains including three Gram-positive (*Bacillus cereus, Staphylococcus aureus*, and *Enterococcus faecalis*) bacteria and three Gram-negative (*Escherichia coli, Klebsiella pneumoniae*, and *Pseudomonas* *aeruginosa*). The antimicrobial activities of the crude extract and the purified flavonoid are shown in Table 3. The recorded level of inhibition has been presented in percentages after an initial calculation of the average instrument readings (± standard deviations). The percentage inhibitions of the samples are presented as follows.

The concentration (strength) of the reference antibiotics were doubled (111 µg/mL) relative to the concentration of the tested samples (55 µg/mL) to test the efficacy of the samples at 50% strength relative to the known antibiotics. From the results (Table 3), the flavonoid was inactive against *B. cereus*, whereas the crude extract showed significant activity (61% inhibition). In [26], the antibacterial activity of natural products isolated from Indian medicinal plants was investigated, and it was reported that the obtained crude extracts exhibited wide antibacterial properties against both Gram-positive and Gram-negative bacteria, including *B. cereus;* however, although the methanolic extract from this study showed high inhibition strength against *B. cereus*, it was inactive against *S. aureus.* The synergistic activity of compounds in the crude might be responsible for the observed level of activities. Volatile oils from *L. flavescens* were found to exhibit strong antimicrobial activities against both Gram-positive *B. cereus* and *S. aureus* due to the high concentration of nerolidol found in the essential oils [2] but showed no activity against Gram-negative *E. coli*.

The reference antibiotic (ampicillin) was inactive against *K. pneumoniae,* however the crude extract showed some activity (23%) against the microbial strain. Gram-positive *S. aureus* was observed to be resistant to the crude extract and the purified flavonoid at the tested concentrations. The crude extract and the pure flavonoid (6-methyltectochrysin) were inactive against *P. aeruginosa* though ampicillin showed 11% inhibition at a concentration of 111 µg/mL.

The activity of the reference standard antibiotic (ampicillin, 111 µg/mL) against the *E. coli* strain was found to be relatively low (6%), indicating a strong level of drug resistance exhibited by the bacterial strain. The flavonoid compound was inactive against the strain of *E. coli,* whereas the crude extract showed positive but low activity (5%).

The crude extract showed 17% inhibition against *E. faecalis* while the flavonoid was almost inactive (2%). Both samples were inactive against *S. aureus* and *P. aeruginosa*. Ampicillin was inactive against *K. pneumonia*, while the crude was about six times (23%) more active than the isolated flavonoid (4%) against the bacteria.

The incidence of drug resistance is still a global burden. The results from this study showed a general resistance exhibited by the Gram-negative strains, which coincidentally have been reported to exhibit higher resistance due to the impermeable outer membrane structure. Unfortunately, conventional treatments have been shown to be less effective in managing microbial infections, mainly due to the increasing drug resistance of these microorganisms, which directly results from drug overdose, negligence and poor infection control practices [27].

Polyphenols are reported to have good antimicrobial activities because they can inhibit biofilm formation, lower adhesion of host ligands, and could neutralize bacterial toxins [28]. Polyphenolic compounds are now being applied as food preservatives [18] due to increasing global appeal for substitution of synthetic preservatives in food. The methanolic crude extract showed such promising potential.

## 3. Materials and Methods

### 3.1. Reagents and Materials

Ethyl acetate, hexane, methanol, silica gel and TLC plates were purchased from Merck (Johannesburg, South Africa). Ascorbic acid, meta-phosphoric acid (MPA), Trolox (6-Hydroxy-2,5,7,8-tetramethylchroman-2-carboxylic acid), gallic acid, fluorescein sodium salt, 2,2′-Azobis (2-amidino propane)dihydrochloride (AAPH), 2,4,6-tripyridyl-s-triazine (TPTZ), 2,2′-azinobis(3-ethyl-benzothiazoline-6-sulfonic acid) (ABTS), iron chloride hexahydrate (FeCl_3_·6H_2_O), chlorogenic acid, sodium acetate, Folin–Ciocalteau reagent, perchloric acid and potassium persulfate were obtained from Sigma-Aldrich (Johannesburg, South Africa).

### 3.2. Spectroscopic Analysis

Column chromatography was done using Silica gel 60 (70–230 and 230–400 mesh sizes, Merck, Johannesburg, South Africa). Thin-layer chromatography (TLC) was performed using TLC plates of silica gel F254 coated on aluminum (Kieselgel 60 F_254_, Merck, Johannesburg, South Africa). Visualization on TLC was achieved under UV light or by spraying the plates with 10% H_2_SO_4_ in ethanol (*v*/*v*). The FTIR spectra of all samples were recorded on a Perkin Elmer UATR Spectrum Two spectrophotometer (PerkinElmer, Inc., Waltham, MA, USA). Samples were scanned over a wavelength range of 4000–400 cm^−1^. The NMR spectra of samples were recorded on Agilent 600 MHz NMR spectrometer (Agilent Technologies, Inc., Santa Clara, CA, USA) using trimethylsilane (TMS) as internal standard. Chemical shifts were recorded in ppm. Mass spectroscopic (ESI-LC/MS) analysis was done on a Bruker AmaZon Ion Trap instrument (Bruker Daltonics, Billerica, MA, USA), with 1 mg of samples. The HPLC instrument was equipped with Dionex^®^ 3000 RS pump, Dionex^®^ WPS-3000RS autosampler and Dionex^®^ DAD-3000RS Diode Array Detector (Thermo Fisher Scientific, Waltham, MA, USA). Separation was effected on Waters^®^ Sunfire C18 reversed phase column (3.5 μm, 4.6 × 150 mm, Waters, Milford, MA, USA) at 30 °C. Samples were injected (injection volume of 10 µL) using the autosampler at a mobile phase multi gradient ratio (A-water, B-Acetonitrile, C-0.1% formic acid) from 2% B to 98% B within 80 min at a flow rate of 0.5 mL/min.

### 3.3. Plant Materials Collection and Extraction

**Figure 4:**
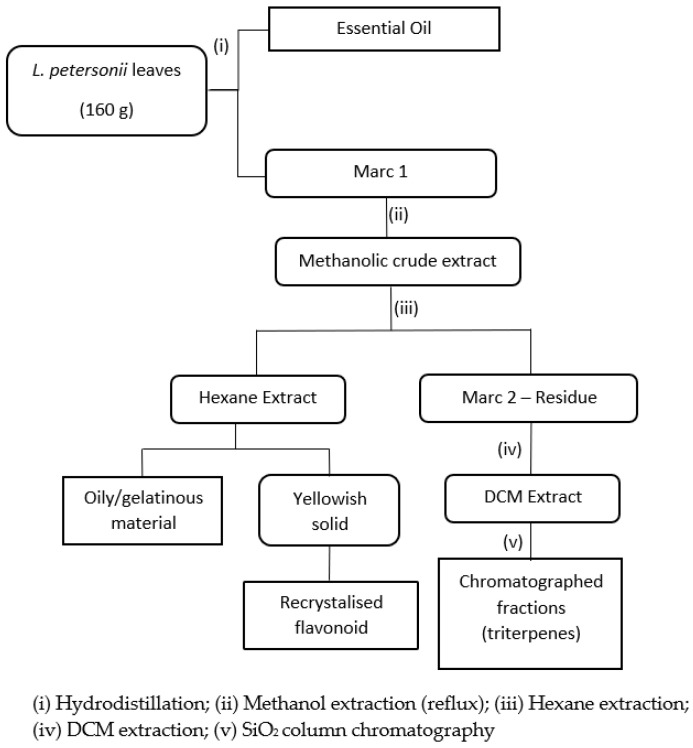
Extraction scheme for isolating natural products from *Leptospermum petersonii* leaves.

Fresh *L. petersonii* leaves were harvested from Durban, South Africa while other defatted (spent) leaves were obtained from an essential oil production facility. The fresh leaves (160 g) were hydro-distilled to extract the essential oil component. This step was skipped for defatted spent leaves obtained from essential oil production facility. The recovered hydro-distilled product was further extracted with hexane to yield 12 mL of the essential oil. The defatted marc (crude) was combined with the spent leaves (100 g) and subjected to solvent extraction using methanol. The resulting methanolic crude extract was partitioned with hexane, and then with dichloromethane (DCM), as represented in Figure 4. The hexane extract was further purified to yield a target flavonoid compound which was characterized using spectroscopic methods. The DCM extract was suspected to contain triterpenes according to preliminary tests and the literature. The bioactivity of the methanolic crude extract before hexane and DCM extraction and the isolated flavonoid were tested in the antioxidant and antimicrobial assays. Figure 4 shows the extraction outline.

### 3.4. Biological Activities

#### 3.4.1. Antioxidant Assays

The antioxidant assays (ORAC, FRAP, and TEAC) of the purified flavonoid and methanolic crude extract were done according to previously reported protocols. In the Oxygen Radical Absorbance Capacity Assay (ORAC) assays, the method of Wu et al. [29] was adapted with slight modifications. Reagents were freshly prepared on the day of analysis to ensure quality control. Samples were dissolved in acetone with the application of sonication where necessary. Calibration standards in the range from 5 to 25 μM were prepared from a standard 500 μM stock solution of Trolox by dilution in phosphate buffer (75 mM, pH 7.4). Fluorescence filters (excitation wavelength of 485 nm and emission wavelength of 538 nm) were employed for the assay. The Fluoroskan ascent plate reader (Thermo Fisher Scientific, Waltham, MA, USA) was equipped with an incubator (working temperature of 37 °C). Fluorescein stock solution was diluted with phosphate buffer to a final concentration of 14 μM per well in a black 96-microwell plate. AAPH (25 mg/mL in phosphate buffer) was then added to each well, resulting in a final AAPH concentration of 4.8 mM in each well. Sample wells contained 12 μL of each sample, in triplicates, while standard and control wells contained 12 μL of Trolox standard and control solutions, respectively. The fluorescence from each well was recorded every 5 min for 2 h. Final ORAC values were calculated using the regression equation *y* = *ax*^2^ + *bx* + *c* between Trolox concentration (μM) and the area under the curve. Results were expressed as micromoles of Trolox equivalents per gram of sample weight.

To determine the Ferric Reducing Antioxidant Power (FRAP) of the samples using the method described by [30]. Each sample (10 μL) was mixed with 300 μL FRAP reagent in a 96-well plate. Samples were added in triplicates to different wells. Control wells contained 10 μL of control per well while ascorbic acid standard wells contained 10 μL of standard per well. The samples were incubated at 37 °C for 30 min, afterwards, the plates were read at a wavelength of 593 nm in a Multiskan Spektrum plate reader (Thermo Fisher Scientific, Waltham, MA, USA). Ascorbic acid (AA) was used as standard. The results were expressed as μmol Ascorbic acid equivalents/100 g sample weights.

In the Trolox Equivalent Antioxidant (TEAC) assays, the method by [31] was employed. To generated ABTS radical cation, 88 µL of freshly prepared 140 mM potassium-peroxodisulphate solution and 5 mL of ABTS solution (7 mM). The solution was mixed and left in a dark enclosure at room temperature for 24 h. The ABTS solution was then diluted with ethanol (1:20 *v*/*v*) to give an absorbance of 1.50 at 734 nm. The samples (dissolved in ethanol) were added to 300 μL ABTS solution in a 96-well clear plate. The Trolox standard wells contained 25 μL of standard solution while the control ones contained 25 μL of the control solutions. Sample wells contained 25 μL of samples in triplicate. The mixtures were incubated at room temperature for 30 min. The recording of the plates was done in a Multiskan Spektrum plate reader (Thermo Fisher Scientific, Waltham, MA, USA). Trolox was used as the standard. Results were expressed as μmol Trolox equivalents/g sample weights.

#### 3.4.2. Antimicrobial Activity

The crude extract and the pure flavonoid were screened to assess the in vitro antimicrobial activity against six multi-drug-resistant pathogenic bacteria, comprising three Gram-positive (*Bacillus cereus* ATCC 10876 (genome-sequenced)*, Enterococcus faecalis* ATCC 51299 and *Staphylococcus aureus* subsp. *aureus* ATCC 33591) and three Gram-negative (*Escherichia coli* ATCC 25922*, Klebsiella pneumoniae* subsp. *pneumoniae* ATCC 700603 and *Pseudomonas aeruginosa* ATCC 27853) microbial strains.

The samples (500 µg/mL in DMSO) were setup in triplicates. The negative control contained 160 µL culture + 20 µL DMSO, while the positive control contained 160 µL culture + 20 µL vancomycin (Gram-positive strains) or ampicillin (for Gram-negative strains). The sterile control contained 50 µL broth + 80 µL sterile distilled water + 50 µL DMSO, while the well for the samples contained prepared sample + 160 µL culture (to a final concentration of 55.6 µg/mL).

The methods of European Committee for Antimicrobial Susceptibility Testing [32] was adapted. Microdilutions of each bacteria and samples were prepared in sterile 96-well microtitre plates in triplicates. The test strains were grown in optimal growth media as indicated previously. After 24 h of growth, the OD_600_ of the culture was determined. The OD_600_ was adjusted to 0.8 for this experiment. The samples and cultures were dispensed into microtiter plates and then incubated for 24 h at the optimal growth temperature of the test strains. 20 µL of 0.25% (*w*/*v*) MTT in PBS was added to each well and the plates were incubated at the optimal growth temperature of the test strains for 3 h. Then, 100 µL DMSO was added to each well and incubated at room temperature for 4 h. The OD of the samples were determined at 570 nm. The levels of microbial inhibition were expressed as a percentage by comparing the OD values for the samples or the antibiotics against the negative control values, expressed as follows

(y − z)/y × 100(1)

where y = OD value for negative control; z = OD value for samples tested.

### 3.5. Statistical Analysis

Statistical analysis for the bioassays was done with GraphPad Prism 5.01 software. The results were given as mean ± standard deviation (SD) of replicate experiments. The *t*-test was used for comparison across data groups. The statistically significant results are those with *p* < 0.05.

## 4. Conclusions

The biological activities of crude extract of *L. petersonii* were compared against the isolated flavonoid, 6-methyltectochrysin which was isolated from the methanolic extracts. The crude extracts showed stronger antioxidant activity (up to 8-fold greater) and higher antimicrobial activity relative to the pure 6-methyltectochrysin. The antimicrobial activities were more improved for Gram-positive bacteria strains than the tested Gram-negative strains. Other reports abound on the biological activities of the essential oils extracted from *L. petersonii* species, which may suggest that the essential oil is the major bioactive component of the plant, especially in antimicrobial assays. This study has, however, shown that spent (defatted) leaves, obtainable in the form of discarded waste from essential oil production factories, possess promising use as natural food preservatives to avoid bacteria growth. Future work may access the strength of these products in the inhibition of enterotoxin secretions that may cause food poisoning. The practice of immediate disposal of spent leaves of the *Leptospermum* after extraction of essential oils may be reconsidered. Since *L. petersonii* is edible, we also suggest the possibility of phytomedicinal formulations or other culinary use of the plants, especially in regions burdened with *B. cereus*.

## Figures and Tables

**Table 1:**
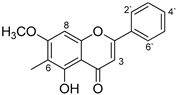
^13^C-NMR spectral data of 6-methyltectochrysin.

Carbon Position	6-Methyltectochrysin
2	163.2
3	105.0
4	183.0
5	160.2
6	105.2
7	163.7
8	95.0
9	154.5
10	103.99
1`	131.71
2`, 6`	126.2
3`, 5`	129.1
4`	131.77
7-OCH_3_	56.0
6-Me	7.7

**Table 2:** Total Antioxidant Capacities of 5-hydroxy-7-methoxy-6methylflavone and *L. petersonii* Crude Extract.

Samples	ORAC Assay (µmol TE/g DW)	FRAP Assay (µmol AAE/g DW)	TEAC (ABTS+) Assay (µmol TE/g DW)
Ascorbic acid (Control)	2567.72 ± 46	-	-
5-hydroxy-7-methoxy-6-methylflavone	48.93 ± 7 ^a^	22.35 ± 4 ^a^	62.1 ± 2 ^a^
*L. petersonii* crude	101.76 ± 8 ^a^	165.51 ± 3 ^a^	191.5 ± 8 ^a^

^a^ Significant difference between the means of the flavonoid and crude extract across various assays (*p* < 0.05).

**Table 3:** Antimicrobial Activities of 6-methyltectochrysin and *L. petersonii* Crude Extract.

Samples/Strains	*B. cereus*	*E. faecalis*	*S. aureus*	*E. coli*	*K. pneumoniae*	*P. aeruginosa*
Positive control ^#^ Vancomycin	71%	14%	27%	-	-	-
^##^ Ampicillin	-	-	-	6%	n.d.	11%
6-methyltectochrysin	n.d.	2%	n.d.	n.d.	4%	n.d.
*L. petersonii* crude	61%	17%	n.d.	5%	23%	n.d.

[antibiotics] = 111 µg/mL [samples] = 55 µg/mL ^#^ Vancomycin = reference antibiotic standard for Gram-positive strains ^##^ Ampicillin = reference antibiotic standard for Gram-negative strains. n.d. = none detected.
